# Antoine Béclère (1856–1939): His Great Contribution to Radiotherapy

**DOI:** 10.7759/cureus.68365

**Published:** 2024-09-01

**Authors:** Panagiotis Sideris, Spyros N Michaleas, Sofia Voidila, Vasileios Mantikas, Ioannis Nikolakakis, Marianna Karamanou

**Affiliations:** 1 Department of Internal Medicine, 417 Army Share Fund Hospital, Athens, GRC; 2 Department of History of Medicine and Medical Ethics, National and Kapodistrian University of Athens School of Medicine, Athens, GRC; 3 Department of Radiology, Health Center of Corinth, Corinth, GRC; 4 Department of Internal Medicine, Gesundheitszentrum Dielsdorf, Dielsdorf, CHE

**Keywords:** charles jacques bouchard, history of medicine, stereoscopic x-ray, radioscopy, radiology

## Abstract

After Wilhelm Conrad Röntgen discovered X-rays in 1895, French physician Antoine Louis Gustave Béclère pioneered the development of radiology in the late 1800s. Béclère recognized the enormous potential of radiation both diagnostically and therapeutically. His radiotherapy techniques quickly gained international renown. In 1897, he founded the world's first radiology teaching lab, the Hospital Radiology Laboratory at Tenon Hospital in Paris. As a hospital physician and researcher, Béclère also had endocrinology, immunology, and virology expertise and published several important papers on various diseases, including many articles on cancer treatment.

## Introduction and background

**Figure 1:**
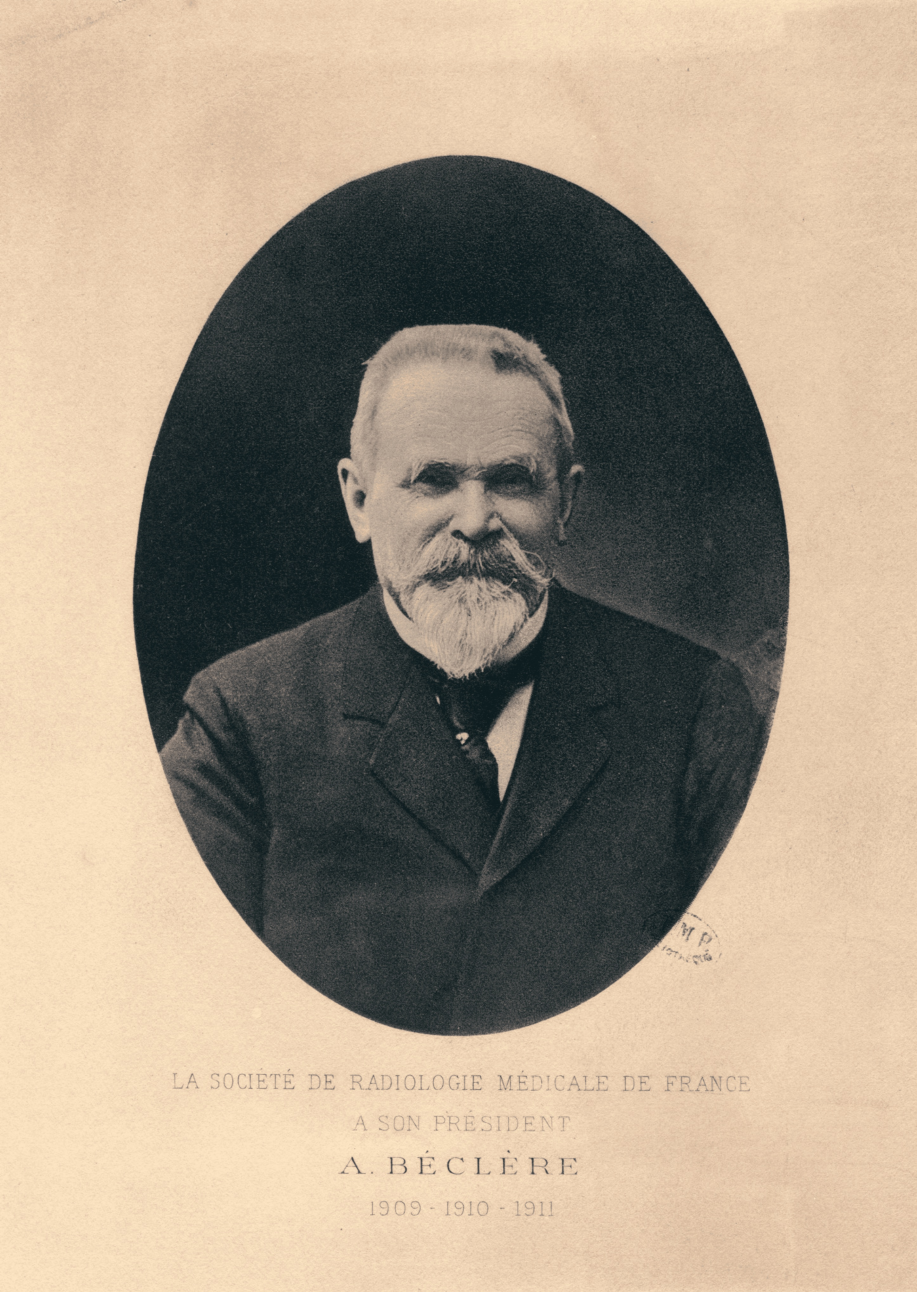
Antoine Béclère (1856-1939) This is an open-source image reproduced under the Creative Commons license. Source: https://www.wikidata.org

Antoine Béclère (1856-1939) was a French physician who pioneered the use of radiotherapy, namely, the use of X-rays in diagnosis and treatment of diseases and injuries (Figure 1). When Wilhelm Conrad Röntgen (1845-1923) discovered X-rays, in 1895, Béclère immediately recognized their substantial diagnostic potential in medicine, stating: “This path seemed to me to be the road that led to the Promised Land, and so I followed it." After observing an instrument known as a “palpateur,” which allowed accurate measurement of the radiation absorbed by the skin, he dedicated himself entirely to radiotherapy. The main purpose of this article is to describe the contribution of his work to medicine and his status as the father of radiology and radiotherapy [1].

## Review

Early works

Béclère was accepted at the Lariboisière Hospital in 1873 and qualified as an intern in 1877 [1,2]. He developed a keen interest in pediatrics, specializing in immunology and writing his doctoral thesis on measles. He excelled in his career and became director of the general medicine clinic at the Tenon Hospital in Paris. After Röntgen’s discovery of the X-ray, French physician and medical researcher Paul Oudin (1851-1923) invented the Oudin coil, a high-voltage electrotherapy technique that was used to treat a variety of conditions. Béclère brought his patients to Oudin’s clinic and was impressed by the treatment the patients received. Therefore, on January 1, 1897, he established the Hospital Radiology Laboratory, the first international radiology school, offering free training to prospective radiologists. The curriculum comprised seven lectures, with training sessions lasting two years [1,2].

Béclère required that all newly admitted patients to the Hospital Radiology Laboratory of Tenon Hospital receive a chest X-ray, creating a pioneering protocol for preventive screening concerning pulmonary tuberculosis detection. Later, in 1897, Béclère presented a series of papers on the radiological diagnosis of chest organs to the Société Médicale des hôpitaux de Paris (Medical Society of the Hospitals of Paris). Béclère believed that although radiography could reveal the existence and location of lesions, it could not identify the nature, or specific pathognomonic nature, of the lesions [3]. This observation has gained critical relevance in modern medicine, where collaboration between radiology and other medical specialties is now ubiquitous in the diagnosis and treatment of illness and injury.

Radiotherapy at Saint Antoine Hospital

Béclère was appointed to the Saint Antoine Hospital on January 1, 1899, where he became known as the founder of medical radiology. He began teaching radiotherapy at Saint Antoine in November 1901, and the hospital featured his course in its promotional materials. He remained at Saint Antoine until 1921 when he became famous for his groundbreaking designs of simple and practical instruments. The below-mentioned instruments were copied and manufactured worldwide, such as the “diaphragm of the iris type” and the “indicateur d’incidence” improved on Hyacinthe Guilleminot’s (1869-1922) tube-bearing frame design [4]. The frame, which carried a screen and a lamp, was suitable for fluoroscopy and for simple stereoscopic radiography of all parts of a person in a standing, sitting, or supine position [3,4]. Béclère also created a frame suitable for fluoroscopy and simple stereoscopic radiography of all planar views, an examination table with a built-in lamp that could move vertically for the examination of a prone person and an examination chair with an integrated cassette apparatus that enabled radiological examination [1,2,5,6]. By introducing radiology into the teaching of medical practice and founding the Hospital Radiotherapy Laboratory, a project done at his own expense, radiotherapy treatment became forever linked to Béclère [1,2,7].

Advancing the field of radiology

Béclère embraced radiotechnology and created, developed, and advanced the field of radiology, proving himself a true clinician [1]. He wisely recognized the importance of fluoroscopy and radiography for examining organs and helped establish radiology as a prominent medical specialty. In 1899, in his article, "Radioscopy and X-rays in Hospitals," Béclère defines X-ray diagnosis as not only a radiographic examination but also radiation therapy. He further posits that X-ray examination must be conducted only by a medical doctor, and thus, every hospital should have an X-ray room and an X-ray laboratory [1-4]. Also, in 1899, he published a manual for the diagnosis of tuberculosis. In 1901, Béclère presented 100 tests of radiological images on glass plates to the Medical Society of Hospitals. When examined in the stereoscope, the relief images revealed osteopathies, arthropathies, acromegaly skulls, and lesions of the lungs, heart, and aorta. That same year, he published a book titled "Röntgen’s Rays and the Diagnosis of Non-Tuberculous Diseases of the Thorax," which he dedicated to Professor Charles Jacques Bouchard (1837-1915), one of his first supporters [1,7].

At the 2nd International Congress of Medical Electrology and Radiology in Bern on September 1, 1902, radiotherapy was no longer seen as an empirical treatment and became officially recognized as a precise science, in great part due to contributions from Béclère and physicians alike [1,3]. Béclère soon turned his interest to digestive tract diseases, a burgeoning specialty in the early 20th century, and began researching esophageal colic [1,8,9]. He eventually presented a paper, "Radiological Control of Surgical Diseases of the Stomach and Intestine," at the 25th French Congress of Surgery, in 1912. Béclère's third book, "Röntgen’s Rays and the Diagnosis of Internal Diseases," was published in 1904, during which he also published two papers on proper dose measurement in radiotherapy. In one paper named, “Means of Protecting the Doctor and Patients from the Morbid Effects of New Radiation,” Béclère proposes the use of protective gloves and goggles, which revolutionized safety procedures for both patients and doctors. In 1905, in his article, “Medical Radiology for Doctors,” Béclère reaffirms that medical radiology, radiodiagnostics, and radiotherapy must be conducted exclusively by medical doctors, and thus theoretical, technical, and clinical instruction should be offered to all medical students in a hospital setting. In 1909, Béclère presented the first X-ray of the appendix and invented a technique for X-raying gallstones [1-3,8].

Many of Béclère's contemporaries built on his techniques and teachings, such as Charles Jacques Bouchard (1837-1915), Edmé Hyacinthe Guilleminot (1869-1922), Georges Maurice Debove (1845-1920), and Jean Alban Bergonié (1857-1925). As previously mentioned, Bouchard was an early supporter who established a radiography and X-ray laboratory in 1898 that was eventually taken over by Guilleminot. Others agreed with Béclère's concerns about how the technology should be used. For example, in 1906, Marie-Emile-Anatole Chauffard (1855-1932), a professor of internal medicine, submitted to the Academy of Medicine of France that the clinical application of X-rays by nonphysicians constitutes medical malpractice [4-7]. One month before his death, Béclère published his final article, “The Treatment of Cancer of the Uterus,” marking the end of 40 decades serving as one of the leading figures in medicine [1,3].

## Conclusions

Antoine Béclère is acknowledged as the father of radiology and radiotherapy. He was among the first physicians to recognize the diagnostic potential of X-rays in diagnosing and treating various conditions. He built on Röntgen’s and Guilleminot’s research and developed revolutionary new techniques in the field, such as screening for tuberculosis using chest X-rays with fluoroscopy. He also helped establish the importance of radiotherapy in diagnosing various conditions, including benign and malignant tumors. Béclère's multitude of published observations highlight his remarkable achievements and unprecedented technical prowess, especially considering the limited means available at the time. His work and legacy helped create the foundation for the vital field of medical radiology.
